# Relationship Between Fracture Toughness and Fracture Mirror in Modern Polymer-Based Dental Composites

**DOI:** 10.3390/jfb16080290

**Published:** 2025-08-12

**Authors:** Nicoleta Ilie

**Affiliations:** Department of Conservative Dentistry, University Hospital, Ludwig-Maximilians-University, Goethestr. 70, D-80336 Munich, Germany; nicoleta.ilie@med.uni-muenchen.de

**Keywords:** resin-based composites, flexural strength, fracture toughness, fracture mirror

## Abstract

The mechanical behavior of dental composites depends on the sample size and stress configuration. This makes it difficult to extrapolate laboratory data to clinical restorations with significant variations in size and geometry. Intrinsic parameters, such as fracture toughness, are therefore of great importance, even if they are less common and more difficult to measure. The aim of this study was to apply principles of fractography and fracture mechanics to exploit the results obtained from a three-point bending test. The objectives include calculating a material-specific constant, validating the experimental findings, and establishing a correlation with fracture toughness. Forty representative composites with wide variation in filler quantity (65–83% by weight and 46.4–64% by volume), type (compact glasses and pre-polymerized), and composition were examined. Fracture toughness/K_Ic_ was evaluated in a notchless triangular prism test. Fracture type, origin, and mirror size were determined on 280 flexural fracture specimens (*n* = 20). The amount of filler strongly influences all measured parameters, with the effect strength varying in the sequence: mechanical work (η_P_^2^ = 0.995), modulus of elasticity (η_P_^2^ = 0.991), flexural strength (η_P_^2^ = 0.988), fracture toughness (η_P_^2^ = 0.979), and mirror constant (η_P_^2^ = 0.965). Fracture surfaces allowed the delineation of the fracture mirror and the application of fracture mechanics approaches. The mirror constant was derived from the radius of the fracture mirror, measured in the direction of constant stress, using Orr’s equation, and correlates well with K_Ic_ (0.81). Larger confidence intervals were observed for the mirror constant data, while for 5 of 14 materials, the mirror constant was overestimated compared to K_Ic_. The overestimation was attributed to the lower refractive index of the urethane methacrylate composition.

## 1. Introduction

Light-curing resin-based composites are among the most popular dental filling materials today. They meet high aesthetic demands and enable a seamless reconstruction of tooth structure in terms of color and, even more importantly and more difficult to mimic, translucency. Further advantages underline their choice in dentistry, as they can be used for minimally invasive dentistry, preserving healthy tooth structures and allowing them to be re-polished or repaired, if necessary, without having to replace the entire restoration. These characteristics are added to high survival rates of composite restorations, approaching 90% in a decade of clinical service [1,2].

The development toward the current high standard and level of maturity was only possible through understanding the relationship between material composition, microstructure, and clinical behavior—a process that is ongoing [1]. Many innovations from other industries have been implemented in dental materials in recent decades, from the introduction of nanotechnology [3] for fillers to new polymerization mechanisms of the organic polymer matrix [4] to intrinsic coloration, which is intended to allow unlimited color adaptation of the material to its environment [5] or bioactivity to prevent the development of secondary caries [6].

The balance between aesthetics and mechanical stability is particularly important for materials used in areas of high mechanical stress, such as posterior restorations [7]. Although there are some guidelines that specify the requirements for a composite for such restorations, for example, a flexural strength of at least 80 MPa [8], there are no clearly defined values for the material properties that can guarantee the long-term clinical longevity of the restoration. For dental materials scientists, it is therefore clear that there is a significant need to establish and define a threshold for appropriate mechanical behavior to help clinicians select an appropriate restorative strategy. While it must be acknowledged that considerable effort has been made to identify these characteristics and define their thresholds, the correlation between laboratory-measured parameters and clinical behavior has been considered moderate to low in most studies to date [9]. The current accepted opinion, supported by studies, is that there is not a single or even a few parameters that can define the clinical behavior but rather a whole series of parameters. Among these, flexural strength and modulus of elasticity [9,10] as well as fracture toughness [7,10] are considered the most important properties for material selection, given that the main reasons for the clinical failure of light-cured composite restorations in the posterior region in recent decades has been bulk fractures, which are responsible for about 70% of filling replacement [2]. In addition, marginal breakdown and clinical fracture are rather related to fracture toughness [10], while clinical wear was mainly connected to the flexural strength [9,10]. Although there is little confirmatory evidence, it is believed that clinical fractures and wear are related to fatigue resistance [10].

The trend toward increased fractures in posterior composite restorations has even intensified in recent decades [1,2]. This is related to increasingly smaller filler sizes and amounts, which impair the mechanical properties of the material, but also to changes in the clinical indication for composite fillings. The clinical success of posterior composite restorations has made practitioners increasingly confident in gradually expanding the indication to include larger, more complex, and multi-surface cavities that are more prone to tooth and restoration fractures [1,2]. Additional factors such as type and location of the restoration, patient age, caries prevalence, bruxism, socioeconomic factors and also the quality and technique of the dentist are being explicitly evaluated and quantified in order to better focus all these effects on their cause and not to distort the effect of the material on the clinical behavior of the material [11].

The comparison of composites tested under different loading conditions in the laboratory aims to simulate the load to which a filling in the oral cavity is subjected [9]. In this context, parameters such as flexural strength, which includes both tensile and compressive stresses during testing, are very frequently determined and are well standardized [8]. Although the flexural strength is measured on a precisely defined sample geometry and under precisely defined conditions to enable reproducible values, the result is valid only for the predefined shape, volume, and geometry of the sample as well as for the test conditions. This calls into question the direct transfer of the results to a dental restoration, whose size and shape vary greatly. More valuable would therefore be parameters that represent intrinsic material properties, i.e., properties that are inherent in a material and do not depend on the factors listed above. An example, therefore, is fracture toughness, which quantifies the ability of the material to resist crack propagation under applied stress. Originating from other industries, various methods to measure fracture toughness have also been introduced for dental materials [12]. The most common are the single-edge notch, compact tensile, double torsion, and short-rod tests [13]. Most of these methods require large sample sizes and are therefore expensive for dental materials. Furthermore, they rely on the application of a sharp pre-crack to initiate fracture, which is complex to implement, technique-sensitive, and prone to error [13].

Based on the above listed reasons, the choice in this study was made on determining fracture toughness by the notch-free triangular prism test (NTP), in which the complex and error-prone pre-crack implementation is no longer necessary. The NTP-test was previously validated for different materials and proved consistent in its outcome with other established tests [14]. Given that flexural strength measurement is the most commonly used method to assess the mechanical properties of composite materials, the idea arose to further exploit these data and complement them with a fractography study. The latter allows the investigation of characteristic fracture features, which can then be quantitatively interpreted to determine fracture toughness as described in Orr theory [15]. In this way, a test that is essentially size-dependent, such as the flexural strength test, should allow the determination of an intrinsic material parameter such as fracture toughness. The aim of the study is therefore to apply principles of fractography and fracture mechanics to the results of a three-point bending test to determine an intrinsic material parameter, the mirror constant, and to verify its correlation with fracture toughness.

Based on the rationale outlined above, two research questions are identified:

1. Can fractography be used to determine the fracture toughness of dental composites?
2. Is the fracture toughness calculated from fractography data validated by the NTP results?

The null hypothesis tested was that after examining a variety of modern dental composites, the fracture toughness determined by quantitative fractography is similar to the outcome of the NTP test.

## 2. Materials and Methods

### 2.1. Materials

A total of forty representative light-cured dental polymer-based composite materials (composites) were randomly selected to represent the modern categories of micro-hybrid, nano, and nano-hybrid composites, with either compact glass fillers and/or pre-polymerized fillers. The filler content varies within a wide range, between 65 and 83% by weight and between 46.4 and 64% by volume. The materials are summarized in Table 1.

### 2.2. Methods

#### 2.2.1. Notchless Triangular Prism (NTP) Test

Fracture toughness K_Ic_ was determined in a notchless triangular prism (NTP) test. Triangular prism specimens (*n* = 15) measuring 6 mm × 6 mm × 6 mm × 12 mm were prepared by compressing the composite between two glass plates separated by a white, split polyacetate (POM) mold, according to the test developed by Ruse et al. [14]. Polymerization occurred in the mold from the top and bottom of the specimens for 20 s (Bluephase^®^ Style, Ivoclar Vivadent, Schaan, Liechtenstein; Irradiance = 1425 mW/cm^2^ measured with a spectrophotometer (MARC: Managing Accurate Resin Curing) system; Bluelight Analytics Inc., Halifax, Canada). After demolding, all three facets of the prism were additionally light-cured for 20 s in an overlapping manner, analogous to ISO 4049:2019 [8]. The samples were stored in artificial saliva at 37 °C for 24 h. Each sample was then mounted in one half of the sample holder to allow for the creation of a small defect using a surgical blade (Ted Pella Inc., Redding, CA, USA). It was then secured in the second half of the sample holder using a specially designed mounting block [16]. Specimens were subjected to tensile loading in the NTP test setup at a crosshead speed of 0.1 mm/min on a universal testing machine (Z 2.5, Zwick/Roell, Ulm, Germany). Force and displacement were continuously monitored and recorded. The maximum force at crack arrest or fracture (F_max_, in N) was used to calculate K_Ic_ in MPa·m^1/2^ using the following equation:

$$K_{Ic}=Y_{min}^{*}\frac{F_{max}}{D\sqrt{W}}$$

where Y_min_* = 28, D = 12 mm, and W = 10.4 mm [16].

#### 2.2.2. Three-Point Bending Test

The flexural strength (FS) and flexural modulus E were assessed in a three-point bending test as described in ISO 4049:2019 [8] with a distance of 20 mm between the supports. For this purpose, 280 (*n* = 20) samples were prepared by compressing the material between two glass plates with polyacetate sheets in between. The plates were separated by a split polyacetate (POM) mold with internal dimensions of 2 mm × 2 mm × 25 mm. The specimens were light-cured for 20 s using the same curing device (Bluephase^®^ Style) as described above and according to ISO 4049:2019 [8]. Similar to the NTP samples, they were all stored in artificial saliva at 37 °C for 24 h. The specimens were loaded to failure in a universal testing machine (Z 2.5 Zwick/Roell) at a crosshead speed of 0.5 mm/min. The force during bending as a function of the beam deflection was measured in the universal testing machine, while the slope of the linear part of the force–deflection diagram was used to calculate the flexural modulus E. The mechanical work was calculated for each individual sample as an integral of the force–displacement diagram.

#### 2.2.3. Quantitative and Qualitative Fractography Analysis

Fracture pattern and fracture origin were determined fractographically using a stereomicroscope (Stemi 508, Carl Zeiss AG, Oberkochen, Germany). The specimens were placed under the microscope with the tensile zone facing the operator. The fractured surface was photographed perpendicularly after vicinal illumination was set up to reveal the characteristic fracture patterns, particularly the fracture mirror and fracture origin [15]. The robustness of vicinal illumination relies on diffuse reflectance, which is less dependent on crack configurations than direct illumination [15]. The first signs of noticeable roughness after the relatively smooth mirror surface were used to determine the mirror boundaries. All fractured surfaces were photographed using a microscope extension camera (Axiocam 305 color, Carl Zeiss AG, Oberkochen, Germany). The fracture origin was identified as either a volume defect (subsurface) or a surface defect located at the edges or corners of the specimens. The radius of the fracture mirror was then measured in the direction of constant stress, parallel to the tensile side of the specimen, from the fracture origin to the mirror boundary. If the fracture origin could not be precisely determined, the diameter of the mirror was measured and then halved to determine the corresponding radius. The radius was measured using the software ImageJ Version 1.53k (U.S. National Institutes of Health, Bethesda, MD, USA) and subsequently used to calculate the mirror constant A using the Orr equation [15].

$$\sigma \sqrt{R}=A$$

With σ = measured flexural strength, R = radius of the fracture mirror, and A = mirror constant.

For this purpose, the strength values σ were plotted against the inverse of the square root of the radius (1/√R), where the slope of the straight line is the mirror constant A. The mirror constant A is then reported for each material with its 95% confidence intervals.

Three samples per fracture type were subsequently selected for scanning electron microscopy (Zeiss Supra 55VP, Carl Zeiss GmbH, Göttingen, Germany).

#### 2.2.4. Field-Emission Scanning Electron Microscopy (Fe-SEM)

The structural appearance of the filler system of each composite was imagined by means of scanning electron microscopy using electron backscatter diffraction (Zeiss Supra 55VP, Carl Zeiss GmbH, Göttingen), with higher atomic order elements appearing brighter. For each of the 14 tested materials, 3 samples were prepared analogously to the samples for the three-point bending test. The samples, which had been stored in artificial saliva for 24 h, were wet ground with silicon carbide paper (p1200, p2500, and p4000 grit, LECO Corporation, Saint Joseph, Michigan, USA) and polished with a diamond suspension (average grain size: 1 µm; Struers, Hovedstaden, Denmark) until the surface was shiny (automatic grinding machine EXAKT 400CS Micro Grinding System EXAKT Technologies Inc., Oklahoma City, OK, USA).

### 2.3. Statistical Analyses

The normal distribution of the data was confirmed by the Shapiro–Wilk test and a parametric approach was used for further analysis. Fracture toughness K_Ic_, flexural strength FS, flexural modulus E, and mechanical work W were compared using one- and multiple-way analysis of variance (ANOVA) and the Tukey post hoc test for significant differences (HSD), setting an alpha risk of 5%. Furthermore, the influence of the filler weight and filler volume on the above-mentioned parameters was investigated using multivariate analysis (general linear model) evaluated on the above-mentioned parameters. Higher values of the partial eta-squared (η_P_^2^) indicate a larger effect on the measured data (SPSS Inc. Version 29.0, Chicago, IL, USA).

## 3. Results

A multifactorial analysis shows a significant (*p* < 0.001) and very strong influence of filler content on the measured properties. The filler content in weight % had a very strong effect on all measured parameters, with the strongest influence on the mechanical work (higher partial eta-squared value, η_P_^2^ = 0.995), followed by modulus of elasticity E (η_P_^2^ = 0.991), flexural strength (η_P_^2^ = 0.988), fracture toughness (η_P_^2^ = 0.979), and mirror constant A (η_P_^2^ = 0.965). The filler volume had a smaller influence than the filler weight, while the greatest influence was exercised on the modulus of elasticity (η_P_^2^ = 0.834) followed in descending order of impact by the mirror constant (η_P_^2^ = 0.777), mechanical work (η_P_^2^ = 0.688), flexural strength (η_P_^2^ = 0.522), and fracture toughness (η_P_^2^ = 0.406).

The results obtained in the conducted tests are presented for all examined materials in detail below.

### 3.1. Notchless Triangular Prism (NTP) Test

**Figure 1:**
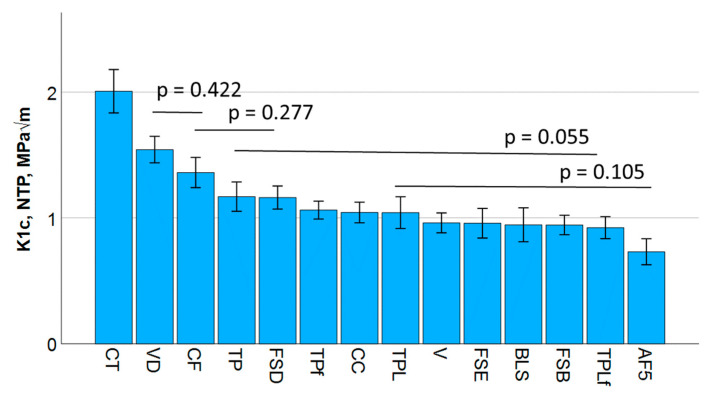
Fracture toughness K_Ic_ determined in a notchless triangular prism (NTP) test. Material abbreviations are shown in Table 1. Data are presented in descending order of K_Ic_ and grouped by bars into statistically significant similar groups.

The one-way ANOVA analysis of the NTP outcome divides the fracture toughness data into five homogeneous groups, ranging from 0.73 MPa√m (AF) to 2.0 MPa√m (CT). The data are summarized in Figure 1. In addition, a Pearson correlation analysis revealed a good correlation of the fracture toughness with the mirror constant (Pearson correlation coefficient = 0.81), the flexural strength (0.60), the flexural modulus (0.51), and the mechanical work (0.47).

### 3.2. Three-Point Bending Test

**Figure 2:**
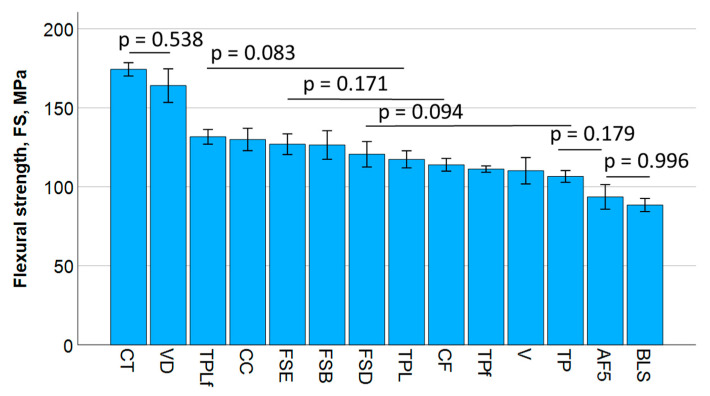
Flexural strength measured in a 3-point bending test. Material abbreviations are shown in Table 1. Data are presented in descending order of strength and grouped by bars into statistically significant similar groups.

The flexural strength of the analyzed materials is shown in Figure 2. Statistically similar data are grouped by bars, and the *p*-values are indicated. One-way ANOVA divides the materials with regard to the flexural strength into six homogeneous subgroups. The highest correlation was found between flexural strength and mechanical work (Pearson correlation coefficient = 0.77), followed by modulus of elasticity (0.63) and fracture toughness (0.6).

**Figure 3:**
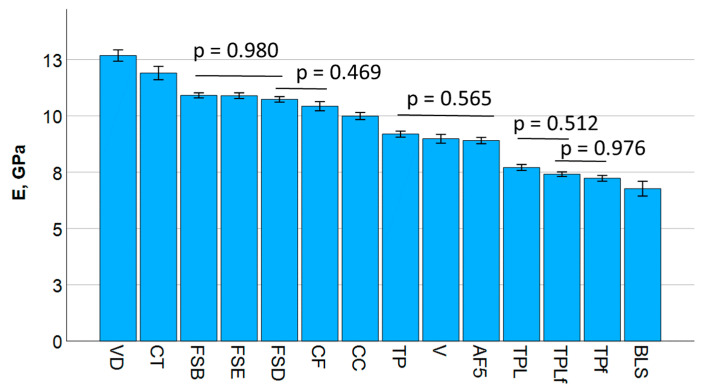
Flexural modulus measured in a 3-point bending test. Material abbreviations are shown in Table 1. Data are presented in descending order of elastic modulus and grouped by bars into statistically significant similar groups.

The results for the elastic modulus are summarized in Figure 3 and divided into nine statistically distinct groups with statistical similarities, grouped by bars. E correlates best with flexural strength (Pearson correlation coefficient = 0.63), followed by fracture toughness (0.51), but not with the mechanical work.

**Figure 4:**
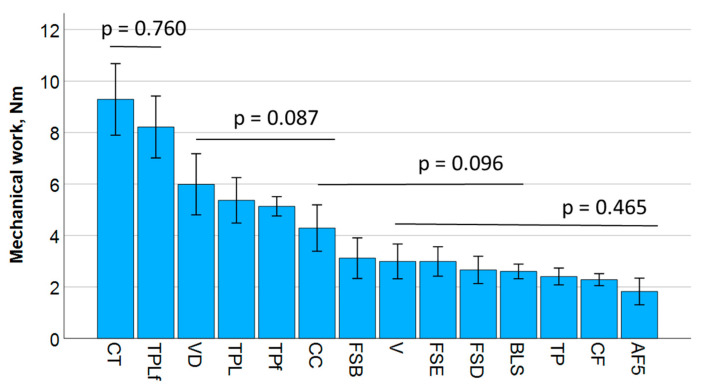
Mechanical work determined in a 3-point bending test. Material abbreviations are shown in Table 1. Data are presented in descending order of mechanical work and grouped by bars into statistically significant similar groups.

The mechanical work was calculated as the integral of the force displacement diagram of the 3-point bending test and refers to the energy that is transferred to the sample by applying force along the measured displacement. The results are summarized in Figure 4 and divide the materials into four different statistical groups. The mechanical work correlates best with flexural strength (Pearson correlation coefficient = 0.77) and fracture toughness (0.47) but not with the elastic modulus.

**Figure 5:**
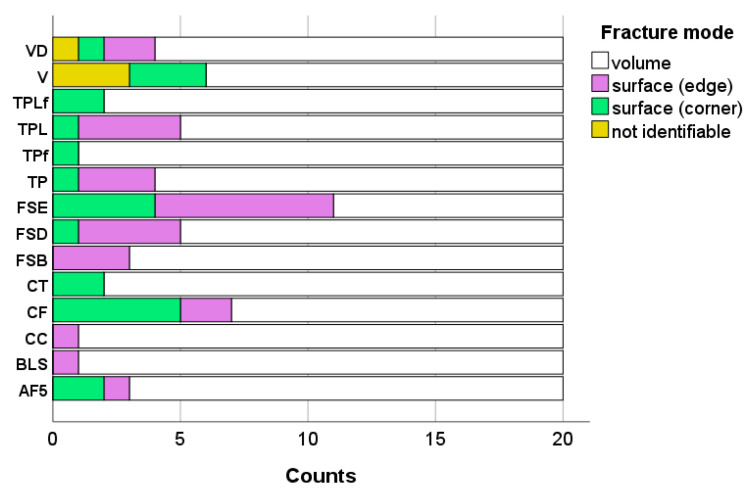
Fracture mode distribution among analyzed materials. Material abbreviations are shown in Table 1.

**Figure 6:**
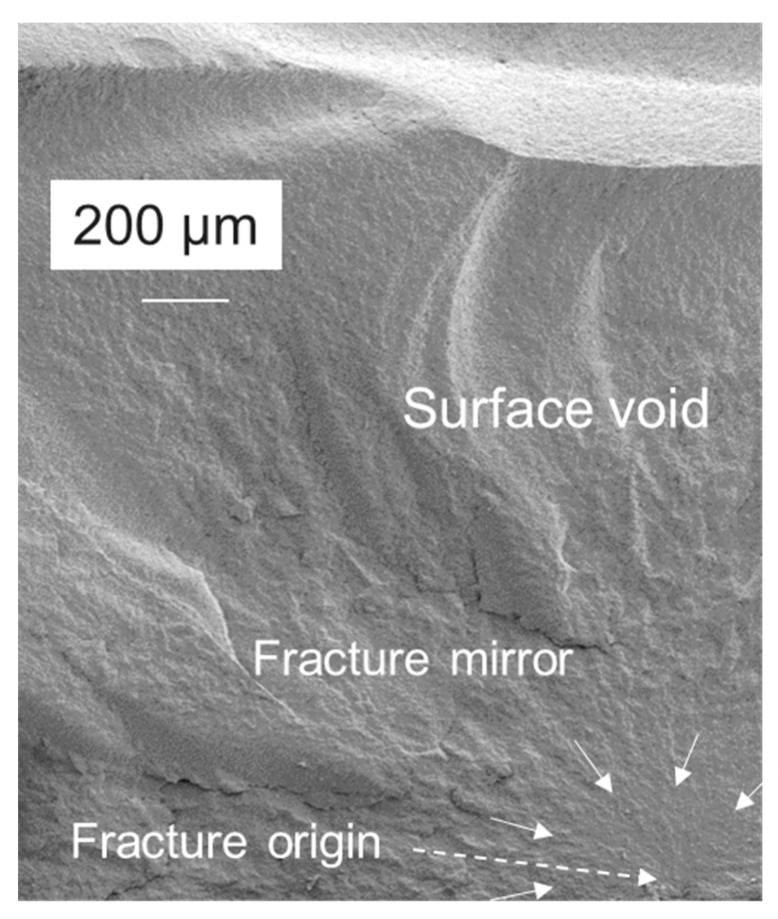
Fractographical analysis of the samples tested in the 3-point bending test exemplified on a subsurface void, located very close to the surface, as fracture origin. Arrows indicate the fracture mirror and mark the defect situated in the tensile zone of the sample loaded in a 3-point bending test.

All samples broken in the 3-point bending test were analyzed fractographically. Failure mode and some representative examples of fracture modes are summarized in Figure 5 and Figure 6. Fractures arise either from a defect below the surface—a void or an agglomeration of fillers—or from a surface defect, usually through a void or a porosity opened during the grinding process.

### 3.3. Quantitative and Qualitative Fractography Analysis

#### 3.3.1. Qualitative Fractography

Qualitative fractography revealed that in the 280 specimens fractured in the 3-point bending test, failures initiated by volume defects are the prevalent failure mode (80.4%) with surface defects accounting for 10.0% (edge) and 8.2% (corner), while 1.4% of the failures were not clearly identifiable. Fracture modes distribution within each material is indicated in Figure 5.

Figure 6 shows representative Fe-SEM images illustrating the most common defect type initiated from a void.

#### 3.3.2. Quantitative Fractography

**Figure 7:**
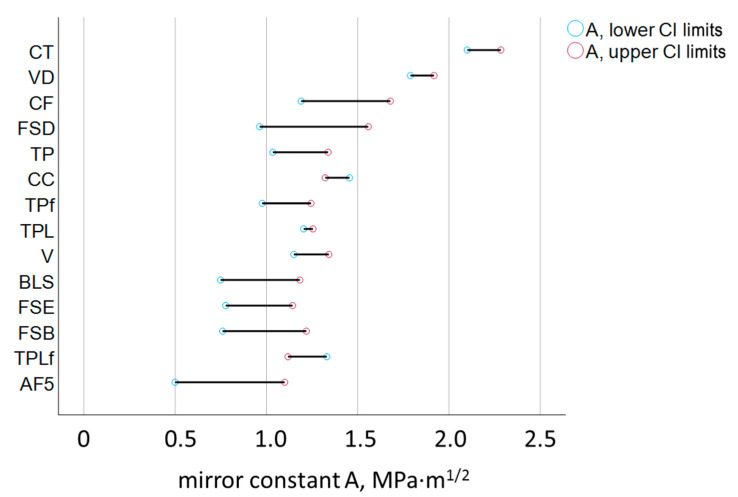
Mirror constant A calculated from the parameters determined in a 3-point bending test. Material abbreviations are shown in Table 1. Data are presented in ascending order of the mirror constant. The 95% confidence interval (CI) is presented with its lower and upper limits.

Each specimen fractured in the three-point bending test was examined fractographically to visualize the fracture mirror and measure the mirror radius. The calculated mirror constant A is shown in Figure 7 and is given with the 95% confidence interval (lower and upper limits are indicated). The Pearson correlation analysis showed a very good correlation between the mirror constant A determined by quantitative fractography and the fracture toughness determined in the NTP test (Pearson correlation coefficient = 0.81).

### 3.4. Scanning Electron Microscopy (SEM)

**Figure 8:**
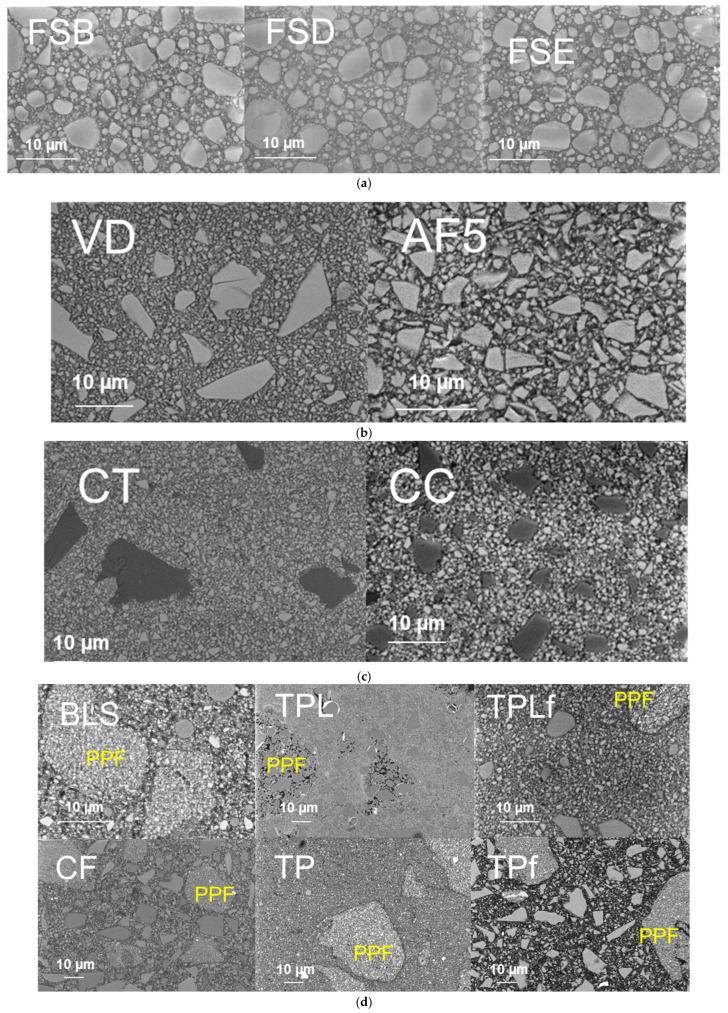
Structural appearance of the filler systems: Fe-SEM-images collected in an electron backscatter diffraction mode enable differentiation between components: polymer appears dark gray; glass filler light-gray to white (elements with higher atomic order). (**a**) Nano composites: FSB = Filtek Supreme XTE Body; FSD = Filtek Supreme XTE Dentin; FSE = Filtek Supreme XTE Enamel. (**b**) Micro-hybrid composites with compact glass fillers: VD = Venus Diamond; AF5 = Admira Fusion 5. (**c**) Micro-hybrid composites with compact glass fillers (light) and homogeneous polymer-based fillers (dark): CT = Charisma Topaz; CC = Charisma Classic. (**d**) Micro-hybrid composites with compact glass fillers and pre-polymerised (PPF) fillers: BLS = Beautifil II LS; TPL = Tetric plus Fill; TPLf = Tetric plus Flow; CF = Cention Forte; TP = Tetric Power Fill; TPf = Tetric Power Flow.

The structural appearance of the filler systems is shown in Figure 8. The images were acquired in backscatter mode, so elements with higher atomic order appear brighter. Figure 8a shows the three nano composites FSB, FSD, and FSE with nano-zirconia and silica fillers, either isolated or clustered into larger micrometer-sized fillers. Figure 8b groups together three characteristic micro-hybrid composites with compact, geometrically irregular, split glass fillers. Large glass fillers above 10 µm can be identified in VD, with medium sizes below 5–7 µm in AFS and very small fillers below 1 µm in V. Another category of micro-hybrid composites is summarized in Figure 8c. In addition to compact, geometrically irregular, cleaved glass fillers as described above, a small number of fillers can also be observed that appear lighter, i.e., have a high polymer content, but are homogeneous. Finally, Figure 8d summarizes composites with regular glass fillers and inhomogeneous pre-polymerized fillers of different size and composition.

## 4. Discussion

Intrinsic material properties are of great importance when it comes to translating laboratory measurements into the clinical behavior of a material placed in cavities of different sizes and geometries. Because dental composites are highly filled polymers, they exhibit pronounced brittle behavior. This was clearly evident in the present study in their fracture surfaces that allow the delineation of the fracture mirror, the smooth radial region surrounding the critical flaw that initiated fracture [17]; the subsequent mist region formed by secondary cracks that fail to propagate due to decreasing energy [18] as the crack velocity increases [19]; and finally the transition into the region of larger radial ridges, the hackle lines with macroscopic crack branching [20]. While these characteristic patterns depend on a variety of parameters, including the size and shape of the critical flaw [19] or additional flaws encountered during crack propagation [21], the critical flaw in the analyzed materials is always located on the tensile side of the specimen loaded in the 3-point bending test, and the fracture propagates toward the neutral axis, leaving a somewhat elongated mirror with an often flared, incomplete shape [17] (Figure 6). In this context, the fracture mirror draws attention to the origin of the fracture, indicating that internal voids and defects, rather than surface defects, were the cause of failure. Volume defects amounted to 80.4%, with many of the surface defects being originally internal voids that were opened during sample grinding. Grinding was deliberately chosen because it not only allows the removal of bulges, protrusions, and other defects that occurred during sample preparation but also corresponds to the clinically procedure for completing a composite restoration.

The incorporation of fillers into the monomer system to create a composite carries the risk of the formation of microdefects such as agglomerates or voids, which can trigger fractures. Voids may already be present in the unpolymerized composite paste but may also have arisen during compaction of the composite paste for sample preparation. On the other hand, surface treatment is sensitive to the material’s microstructure, so the occurrence of surface defects may in fact be related to filler size and inherent material roughness. Another aspect to consider is the quality of silanization, since fillers that are weakly embedded in the polymer matrix, as well as pre-polymer fillers, which are known to have a weaker bond to the organic matrix than compact fillers, can easily be pulled out during grinding, leaving a defect large enough to initiate fracture. The 14 composites analyzed illustrate the great diversity of dental composite microstructure. However, their fracture mode distribution, summarized in Figure 5, does not allow a clear assignment of surface defects to the aforementioned aspects. Larger filler sizes or materials with pre-polymerized fillers do not appear to exhibit increased surface defects, which again demonstrates that various effects operate simultaneously in such complex materials.

The flexural strength data for all materials significantly exceeded the ISO standard requirement of 80 MPa. This fact can distort the right decision for a material, as particularly for flowable, low-filled materials, the high polymer content leads to greater beam deflection during testing, resulting in a longer failure time and increased flexural strength. In contrast, the elastic modulus is directly related to the proportion of inorganic fillers, placing the tested low-filled flowable materials significantly at the bottom of the rankings. In addition, for materials with pre-polymer fillers, the total filler quantity indicated does not correspond to the quantity of inorganic filler, since this also includes the polymer phase of the filler, which has a lesser impact on the elastic modulus. Therefore, when selecting a suitable material, flexural strength data must always be accompanied by elastic modulus data. There is currently no threshold value for the modulus of elasticity when selecting materials for posterior restorations. However, in order to withstand elastic deformation, a high modulus should be chosen. Other factors such as size, shape, distribution, and the nature of the filler’s bond to the organic matrix may act antagonistically on the mechanical parameters. Filler size is particularly important for the primary dependence of mechanical properties such as wear [22,23]. In contrast, the filler shape influences flexural strength and elastic modulus, with irregular fillers exercising a positive effect compared to spherical fillers at a given volume fraction [24]. The incorporation of fillers into a polymer matrix also hinders the formation, growth, and propagation of cracks or requires more energy for this resulting in higher fracture toughness values. The material VD tested in the present study was the composite with the highest filler content in both weight and volume percent, which was indeed clearly reflected in the highest elastic modulus. Due to the direct relationship between elastic modulus and fracture toughness, the highest fracture toughness would be expected for VD, but CT exceeded it. This behavior needs to be related to two other aspects: the smaller filler size in the CT, which allows for a consistently higher filler–matrix interface, thus favoring crack deviation and energy dissipation, and the incorporation of pre-polymerized fillers, even if only in a small proportion of 1%. These pre-polymerized fillers offer additional opportunities to increase fracture toughness, as the bond of the pre-polymerized filler to the matrix is weaker compared to compact fillers, which can lead to blunting of the crack tip and thus a delay in crack growth. The detachment of such regions can also delay crack growth and dissipate energy due to the reduced driving force at the crack tip.

The fracture mirror not only allows the identification of the fracture origin but also provides information about the magnitude and distribution of the stresses that caused the fracture [12]. One century ago, it was empirically observed that the smaller the fracture mirror, the greater the stress at the point of origin. Conversely, larger mirrors indicate lower fracture stress and a large defect that initiated fracture. Orr expressed later this relationship mathematically by relating the applied stress to the inverse of the square root of the mirror radius, with the slope of this relationship representing a constant defined as the mirror constant [15]. The relationship has been verified for glasses, polycrystalline ceramics of various compositions and microstructures, as well as single crystals [19]. The principle of fracture mechanics has already been successfully applied to dental materials [12]. This was primarily performed in the field of ceramics [12] but has increasingly been applied to brittle CAD/CAM composites and light-curing composites [15,25].

The fracture toughness, a material property that quantifies the resistance of a material to crack propagation under applied stress, and the mirror constant, derived from the size of the fracture mirror, are in good agreement in the present study and show a high correlation. This validates and encourages the use of quantitative fractography in brittle dental composites. However, it is noticeable that the data obtained by quantitative fractography have a significantly higher confidence interval (Figure 7), which is due to the higher risk of error in the optical evaluation of the mirror boundaries. These were delineated as the first signs of noticeable roughness after the relatively smooth mirror surface. Although standardized illumination techniques and a trained operator were involved, the detection of the transition to the mist zone of the fractured surfaces is prone to individual perception and interpretation. Improvements can be achieved by involving multiple operators in the assessment [12], by averaging the results and thus reducing operator-related errors. However, the ranking of the materials was comparable and the determined fracture toughness and mirror constant values were statistically similar for 9 of the 14 materials tested. Quantitative fractography revealed a slight overestimation of the mirror constant compared to K_Ic_ for only 5 materials—V, VD, CC, TPL, and TPLf. This means that the mirror radius was overestimated for these materials. A first plausible influencing factor would be the intrinsic roughness of the materials. Higher roughness can lead to a less smooth and shiny mirror and impair the accuracy of the delineation of the fracture mirror from the mist region. After a detailed analysis of the microstructure, this assumption is rejected because the above five materials actually have different compositions and exhibit strong differences in intrinsic roughness due to the different size and type of filler. V and VD are composites with compact glass filler, either the smallest or largest filler size of the tested materials. CC also contains a small amount of pre-polymerized filler, while TPL and TPLf are composites with a mixture of compact and pre-polymerized filler. The amount of filler cannot be used as an explanation in this context either, since the scatter of the filler amount between the five materials is also very high (Table 1), which does not allow any differentiation with regard to the materials for which the radius was not overestimated. The lowest common denominator among the five materials appears to be the composition of the organic matrix, which is based on urethane methacrylate in all five materials. In contrast, the remaining tested materials are predominantly based on bis-GMA. Differences in optical properties, such as the lower refractive index of the organic matrix rich in urethane methacrylate compared to those based on bis-GMA, could therefore be taken into account, making the transition between the two regions more difficult to detect.

The determined material parameter, the mirror constant, and its relationship to fracture toughness can be applied in clinical fractography. In the case of a fractured composite restoration, the measurement of the fracture mirror size and knowledge of the material used for the restoration can be correlated with the mirror constant to provide an indicator of the stress the filling was subjected to during fracture. This information helps estimate the stress on the filling in the patient’s mouth and assess the resilience of a filling. This enables better and more efficient future treatment planning, as fractures are the most common reason for replacing a composite filling.

## 5. Conclusions

The present study shows that fractography and fracture mechanics can be used to determine a material parameter, the mirror constant, in polymer-based dental composites and validates this result by demonstrating a good correlation with fracture toughness. Minor deviations were observed for a small subset of materials with lower refractive indices, where the boundary between the mirror and mist zones was slightly overestimated.

## Figures and Tables

**Table 1:** Analyzed composites: abbreviation (code), brand, manufacturer, shade, batch, and filler quantity (weight and volume percent) according to the manufacturer’s specifications. The exposure time for all materials was 20 s (violet-blue LED, Bluephase Style).

Code	Brand	Manufacturer	Shade	LOT	Monomer	Filler wt./vol.%
TPf	Tetric^®^ PowerFlow	Ivoclar-Vivadent, Scaan, Liechtenstein	^IV^A	Z05FP4	Bis-EMA Bis-GMA, TDM	68.2/46.4
TPLf	Tetric plus Flow		A3 plus	YM0210	-	65/50–51
TP	Tetric^®^ PowerFill		^IV^A	Z04FD5	Bis-GMA, Bis-PMA, UDMA, Bis-EMA, TDM	76–77/53–54
TPL	Tetric plus Fill		A3 plus	YM0202	-	70/51–52
CF	Cention^®^ Forte		A3	Z05DPZ	UDMA, DMA	-/58–59
FSD	Filtek^TM^ Supreme XTE Dentin	Solventum, Maplewood, MN, U.S.	A3	N940010	Bis-GMA, UDMA, TEGDMA, bis-EMA	78.5/53
FSE	Filtek^TM^ Supreme XTE Enamel		A3	N960393	Bis-GMA, UDMA, TEGDMA, bis-EMA	78.5/53
FSB	Filtek^TM^ Supreme XTE Body		A3	N959150	Bis-GMA, UDMA, TEGDMA, bis-EMA	78.5/53
AF5	Admira^®^ Fusion 5	VOCO, Cuxhaven, Germany	A2	2345285	ORMOCER	83/-
BLS	Beautifil II LS	Shofu, Kyoto, Japan	A3	051813	UDMA; Bis-MPEPP; Bis-GMA; TEGDMA	82.9/-
CC	Charisma Classic	Kulzer, Hanau, Germany	universal	K010710	Bis-GMA, UDMA, TEGDMA	77/61_t_(60)_i_
CT	Charisma Topaz		translucent	K010028	TCD-urethane UDMA TEGDMA	75/59_t_(58)_i_
V	Venus		universal	K010518	Bis-GMA TEGDMA	78/59
VD	Venus Diamond		opalescent	K010035	TCD-urethane UDMA TEGDMA	81/64

Abbreviations: wt % = percent by weight; vol % = percent by volume; Subscript t = total filler amount; Subscript i = inorganic filler amount; “-” = no information available. Bis-GMA = bisphenol A glycol dimethacrylate; TEGDMA = Triethylene glycol dimethacrylate; UDMA = Urethane dimethacrylate; TCD-urethane = 2-propenoic acid, (octahydro-4,7 methano-1H-indene-5-diyl) bis (methyleneiminocarbonyloxy-2,1-ethanediyl) ester; Bis-EMA = etoxilated bisphenol A glycol dimethacrylate; TDM = tricyclodocane dimethanol dimethacrylate; DMA= dimethacrylate.

## Data Availability

The original contributions presented in the study are included in the article, further inquiries can be directed to the corresponding author.

## References

[1] Alvanforoush N., Palamara J., Wong R.H., Burrow M.F. (2017). Comparison between published clinical success of direct resin composite restorations in vital posterior teeth in 1995–2005 and 2006–2016 periods. Aust. Dent. J..

[2] Heintze S.D., Loguercio A.D., Hanzen T.A., Reis A., Rousson V. (2022). Clinical efficacy of resin-based direct posterior restorations and glass-ionomer restorations—An updated meta-analysis of clinical outcome parameters. Dent. Mater..

[3] Zhang M., Su Q., Diao K., Bi W., Yan Z., Luo Y., Ren G. (2025). Preparation and characterization of mechanical properties of boron nitride nanosheets (BNNSs)-reinforced resin-matrix ceramics for dentistry. Dent. Mater..

[4] Dasari S., Kasilingam T., Bhatt P., Singh M.S., Kumar S. (2025). RAFT Polymers-Capped Lanthanide-Doped Nanoparticles via “One-Pot” Aminolysis/Thiolate-Grafting. Macromol. Rapid Commun..

[5] Gasparik C., Pérez M.M., Ruiz-López J., Ghinea R.I., Dudea D. (2025). The color of natural teeth: A scoping review of In-Vivo studies. J. Dent..

[6] de Carvalho L.F., Gimenes E.S.M., Barboza A.D.S., Badaró M.M., Stolf S.C., Cuevas-Suárez C.E., Lund R.G., Ribeiro de Andrade J.S. (2025). Effectiveness of bioactive resin materials in preventing secondary caries and retention loss in direct posterior restorations: A systematic review and meta-analysis. J. Dent..

[7] Hill T.J., Stevens C.D. (2025). Fracture Toughness of Esthetic Pressed and Milled Restorative Materials. Int. J. Periodontics Restor. Dent..

[8] (2019). Dentistry—Polymer-Based Restorative Materials.

[9] Ferracane J.L. (2013). Resin-based composite performance: Are there some things we can't predict?. Dent. Mater..

[10] Heintze S.D., Ilie N., Hickel R., Reis A., Loguercio A., Rousson V. (2017). Laboratory mechanical parameters of composite resins and their relation to fractures and wear in clinical trials—A systematic review. Dent. Mater..

[11] Wierichs R.J., Kramer E.J., Meyer-Lueckel H. (2020). Risk Factors for Failure of Direct Restorations in General Dental Practices. J. Dent. Res..

[12] Jodha K.S., Salazar Marocho S.M., Mecholsky J.J., Lirette S.T., Duan Y., Griggs J.A. (2025). Comparing four different methods of measuring fracture toughness and its relationship with fractal dimension in a polycrystalline (3Y-TZP) dental ceramic. Dent. Mater..

[13] Ilie N., Hilton T.J., Heintze S.D., Hickel R., Watts D.C., Silikas N., Stansbury J.W., Cadenaro M., Ferracane J.L. (2017). Academy of Dental Materials guidance—Resin composites: Part I—Mechanical properties. Dent. Mater..

[14] Ruse N.D., Troczynski T., MacEntee M.I., Feduik D. (1996). Novel fracture toughness test using a notchless triangular prism (NTP) specimen. J. Biomed. Mater. Res..

[15] Quinn G.D. (2007). Guidelines for measuring fracture mirrors. Ceram. Trans..

[16] Ilie N., Ruse N.D. (2019). Shear bond strength vs interfacial fracture toughness—Adherence to CAD/CAM blocks. Dent. Mater..

[17] Johnson J., Holloway D. (1966). On the shape and size of the fracture zones on glass fracture surfaces. Philos. Mag..

[18] Yoffe E.H. (1951). LXXV. The moving griffith crack. Lond. Edinb. Dublin Philos. Mag. J. Sci..

[19] Mecholsky J., Rice R., Freiman S. (1974). Prediction of fracture energy and flaw size in glasses from measurements of mirror size. J. Am. Ceram. Soc..

[20] Kirchner H.P., Conway Jr J. (1987). Criteria for Crack Branching in Cylindrical Rods: II, Flexure. J. Am. Ceram. Soc..

[21] Bansal G.K., Duckworth W.H. (1977). Fracture stress as related to flaw and fracture mirror sizes. J. Am. Ceram. Soc..

[22] Lang B.R., Jaarda M., Wang R.F. (1992). Filler particle size and composite resin classification systems. J. Oral Rehabil..

[23] Lawson N.C., Burgess J.O. (2015). Wear of nanofilled dental composites at varying filler concentrations. J. Biomed. Mater. Res. B Appl. Biomater..

[24] Sakai T., Li H., Abe T., Yamaguchi S., Imazato S. (2021). Multi-scale analysis of the influence of filler shapes on the mechanical performance of resin composites using high resolution nano-CT images. Dent. Mater..

[25] Ghelbere R., Ilie N. (2023). Validation of the Orr theory in dental resin-based composites: A fractographic approach. J. Mech. Behav. Biomed. Mater..

